# Depressive Symptoms and Associated Psychosocial Factors among Adolescent Survivors 30 Months after 2008 Wenchuan Earthquake: A Follow-Up Study

**DOI:** 10.3389/fpsyg.2016.00467

**Published:** 2016-03-30

**Authors:** Xuliang Shi, Nancy X. Yu, Ya Zhou, Fulei Geng, Fang Fan

**Affiliations:** ^1^School of Psychology, Center for Studies of Psychological Application, and Key Laboratory of Mental Health and Cognitive Science of Guangdong Province, South China Normal UniversityHong Kong, China; ^2^Department of Applied Social Sciences, City University of Hong KongHong Kong, China

**Keywords:** depressive symptoms, adolescent survivors, follow-up study, earthquake, negative life events, social support, resilience

## Abstract

**Purpose:** This study longitudinally investigated the changes of depressive symptoms among adolescent survivors over 2 years and a half after the 2008 Wenchuan earthquake in China, as well as the predictive effects of demographic characteristics, earthquake exposure, negative life events, social support, and dispositional resilience on the risk of depressive symptoms at two time points after the earthquake.

**Methods:** Participants were 1573 adolescent survivors (720 males and 853 females, mean age at initial survey = 15.00 ± 1.26 years), whose depressive symptoms were assessed at 6 months (T_6m_) and 30 months (T_30m_) post-earthquake. Data on demographics, earthquake exposure, and dispositional resilience were collected at T_6m_. Negative life events and social support were measured at T_6m_ and 24 months (T_24m_) post-earthquake.

**Results:** The prevalence rates of probable depression, 27.5 at T_6m_ and 27.2% at T_30m_, maintained relatively stable over time. Female gender was related with higher risk of depressive symptoms at both T_6m_ and T_30m_, while being only child could only predict higher risk of depressive symptoms at T_30m_. Negative life events and social support at T_6m_, as well as earthquake exposure, were concurrently associated with increased risk of depressive symptoms at T_6m_, but not associated with the risk of depressive symptoms at T_30m_, while negative life events and social support at T_24m_ could predict depressive symptoms at T_30m_, all of which suggested that these variables may have strong but short-term effect on adolescents’ depressive symptoms post-earthquake. Besides, dispositional resilience was evidenced as a relatively stable negative predictor for depressive symptoms.

**Conclusions:** These findings could inform mental health professionals regarding how to screen adolescent survivors at high risk for depression, so as to provide them with timely and appropriate mental health services based on the identified risk and protective factors for depressive symptoms.

## Introduction

Depression is a highly prevalent psychiatric disorder following natural or man-made disasters, especially among children and adolescents (Thienkrua et al., 2006; Gershoff et al., 2010; Fan et al., 2011; Lai et al., 2013; Karam et al., 2014). Adolescents who suffer from depression are more inclined to have interpersonal and academic difficulties (Segrin, 2000; Fröjd et al., 2008; Kochel et al., 2012; Verboom et al., 2014).

As the literature, the prevalence rates of depression among children after natural disasters range from 7.5 to 44.8% (Tang et al., 2014). However, most of the prior studies were cross-sectional and estimated depression prevalence at only one time point. Only few studies have been conducted to longitudinally investigate the changes of depression prevalence among children and adolescents exposed to natural disasters (Thienkrua et al., 2006; Liu et al., 2011; Jia et al., 2013; Ye et al., 2014). For example, Liu et al. (2011) studied 330 children exposed to the Wenchuan earthquake and reported that 14.5 and 16.1% of the sample showed depressive symptoms at 6 and 12 months after the disaster, respectively. Thienkrua et al. (2006) investigated 371 children at 2 and 9 months following a tsunami and estimated the rates of depressive symptoms to be about 10% at both time points. However, these findings are limited by a short follow-up duration of no more than 2 years after a natural disaster, which leaves unclear the changes of depression prevalence among adolescent survivors in a longer time course.

Numerous studies have been conducted to identify predictors of depression and other mental health problems in the aftermath of disasters (Cox et al., 2008). Exposure to the disaster *per se* and stressors caused by the disaster (e.g., negative life events) may play an important role in developing psychopathology (Maes et al., 2001; Boscarino and Adams, 2009; Ye et al., 2014; Fan et al., 2015). Social support has also been commonly observed to be a crucial factor that may buffer the adverse impacts of disasters on persons’ mental health status (Paykel, 1994; Galambos et al., 2004; Pietrzak et al., 2009; Fan et al., 2015). Another repeatedly documented factor is dispositional resilience, a personality trait that enables a person to cope successfully with disasters or other stressors and consistently maintain a healthy psychosocial functioning (Pietrzak et al., 2009; Green et al., 2010; Min et al., 2012). However, most of the existing studies have used a cross-sectional design and merely examined the concurrent associations between these factors and the risk of depression (Wang et al., 2012; Lai et al., 2013; Xu et al., 2013; Pan et al., 2015). Only few studies have investigated the longitudinal predictive validities of the abovementioned factors on a person’s post-disaster depression. For example, Ye et al. (2014) found that the effect of earthquake exposure on survivors’ depressive symptoms tended to abate with time. In a prospective study among 1300 black and white female adolescents, Franko et al. (2004) reported that initial negative life events (especially interpersonal trauma) could predict the onset of depression 5 years later. These studies have mostly concerned with the effects of disaster exposure and other related stressors. It remains relatively unclear whether and the extent to which the predictive validities of social support and dispositional resilience on post-disaster depression would change with the passage of time.

With respect to the drawbacks of previous studies, the present study longitudinally investigated depressive symptoms among adolescent survivors over a period of 2 years and a half in the aftermath of the Wenchuan earthquake. Our specific purposes were to: (1) estimate and compare the prevalence rates of probable depression at 6 and 30 months after the earthquake; (2) and examine the predictive effects of earthquake exposure, negative life events, social support, and dispositional resilience on the risk of depressive symptoms at both time points.

## Materials and Methods

### Participants and Procedure

Participants were 1573 adolescents exposed to the Wenchuan earthquake. They were selected from two high schools in Dujiangyan City which is about 20 kilometers away from the epicenter and was one of the ten worst affected areas in the earthquake. Both selected schools are public schools with enrolled students of various socio-economic backgrounds. Among the 1573 adolescents, 45.8% were males and 82.8% were the only child in their families. 12.8% of them reported family members being killed or missing, 42.3 and 21.5%, respectively, reported severe house damage and property loss, and 61.7% reported direct witness of tragic scenes during the earthquake.

Depressive symptoms of 1573 participants were initially assessed at 6 months (T_6m_), and then at 30 months (T_30m_) following the earthquake. Demographic characteristics (including gender, age, and sibling number), severity of earthquake exposure, negative life events, social support, and dispositional resilience were measured at T_6m_. Because negative life events and social support may change with time, these variables were also measured at 24 months (T_24m_) following the earthquake. Self-reported measures were distributed to participants by psychological professionals in classroom settings and retrieved upon completion. Ethics approval was granted by the Human Research Ethics Committee of South China Normal University. Signed informed consent from both participants and their parents was obtained before the initial assessment was conducted. Participants were informed that they could withdraw from the study at any time.

### Measures

#### Severity of Exposure to Earthquake

Severity of earthquake exposure was measured using four items: casualties among family, house damage, property loss other than house damage, and direct witness of tragic scenes. The first item had five choices: 1 = death of family members; 2 = disappearance of family members; 3 = severe injury of family members; 4 = moderate injury of family members; 5 = none of the above. The other three items were rated on a 5-point scale ranging from 1 (the highest level of exposure) to 5 (the lowest level of exposure).

#### Depression Self-Rating Scale for Children (DSRSC)

Depressive symptoms were measured by the Depression Self-rating Scale for Children (DSRSC) (Birleson, 1981) at T_6m_ and T_30m_. This scale consisted of 18 items, each rated on a 3-point scale ranging from 0 (never) to 2 (mostly). A higher total score indicates severer depressive symptoms. This scale has demonstrated good psychometric properties in Chinese children and a cutoff score of 15 has been recommended for identifying probable clinical depression (Su et al., 2003). In our study, the Cronbach’s alpha values were, respectively, 0.88 and 0.84 at T_6m_ and T_30m_.

#### Adolescent Self-Rating Life Events Checklist (ASRLEC)

Adolescent Self-Rating Life Events Checklist (ASRLEC) (Liu et al., 1997) was used to evaluate negative life events at T_6m_ and T_24m_. It contains 26 items assessing various domains of life events within a timeframe of past 6 months, including interpersonal conflicts, academic pressure, family conflicts, punishment by guardians or teachers, humiliation, monetary loss, health problems, and death or illness of family members. Each item was rated on 5-point scale ranging from 1 (the event never happened) to 5 (the event happened with very strong impact). This scale has shown good reliability and validity among Chinese adolescents (Liu et al., 1997). In our study, Cronbach’ alpha values were, respectively, 0.87 and 0.84 at T_6m_ and T_24m_.

#### Social Support Rate Scale (SSRS)

Social support was measured using the Social Support Rate Scale (SSRS) (Xiao, 1994) at T_6m_ and T_24m_. It is a 10- item scale with three subscales: objective support (e.g., Who or which organizations have ever helped you when you were in need”), subjective support (e.g., “How many close friends did you have to help you”), and utilization of support (e.g., “How frequently did you take part in collective activities”). Objective support refers to actual support a person received in the past time. Subjective support refers to a person’s perception of his/her own social resources. Utilization of support reflects a person’s tendency to actively seek and utilize social support (Ke et al., 2010). A total score was calculated by adding up item scores. Satisfactory psychometric properties of SSRS have been described in a Chinese sample (Xiao, 1994). In our study, Cronbach’s alpha values were, respectively, 0.89 and 0.94 at T_6m_ and T_24m_.

#### Resilience Scale (RS)

Dispositional resilience was measured using the Chinese version of Resilience Scale (RS) (Wagnild and Young, 1993). We only measured resilience at T_6m_, because this construct has usually been considered as a personality trait that may be stable across time and circumstances (Rabkin et al., 1993; Wagnild, 2009; Lei et al., 2012). The scale consists of 25 items, clustering into two dimensions: personal competence (e.g., “I can get through difficult times because I have experienced difficulty before”) and acceptance of self and life (e.g., “My life has meaning”). Each item was rated on a 7-point Likert scale (1 = disagree, to 7 = agree). The total score ranges from 25 to 175, with a higher total score indicating a higher level of dispositional resilience. The scale has demonstrated satisfactory reliability and validity in Chinese adolescents (Li et al., 2010). In our study, the Cronbach’s alpha was 0.90.

### Data Analysis

Of the 1573 participants, 8 and 538, respectively, had missing data on the measurement for depressive symptom at T_6m_ and T_30m_. *T*-test was used to compare the difference in depressive symptoms at T_6m_ between participants with complete data on the measurement for depressive symptoms at both time points (*n* = 1027) and those who had missing data at either time point (*n* = 546). There was no significant difference between two groups (*t* = 1.09, *p* = 0.28), suggesting that the attrition was unrelated to participants’ initial depressive symptoms and the data were missing at random (MAR) (Rubin, 1976). So the expectation-maximization algorithm was used to deal with missing data.

As suggested by existing literature (Su et al., 2003), a cutoff score of 15 on the DSRSC was used in our analysis for identifying yes/no of depression. Four items of earthquake exposure were included in the analysis as categorical variables. Because some responses of the four items had very few participants, the first item was recorded into three categories reflecting family member injured or killed/missing (0 = no, 1 = injured, 2 = killed/missing), the second and third items were also recorded into three categories reflecting severity of house damage and property loss (0 = no, 1 = medium, 2 = severe), and the last item of “witnessing tragic scenes or not” was recorded into two categories (0 = no, 1 = yes). Moreover, because two items of the ASRLEC (“death or illness of a family member”) overlap with the first item of earthquake exposure, they were excluded when calculating the total score of negative life events at T_6m_.

Independent-samples *t*-tests were used in current study. Moreover, multivariate logistic regression analyses were conducted to examine the predictive effects of demographics, earthquake exposure, negative life events, and dispositional resilience on the occurrence of depression at T_6m_ and T_30m_. All predictors were entered into a regression model in one block. For the model of depressive symptoms at T_30m_, the potential cofounding effect of depressive symptoms at T_6m_ was controlled for. A value of *p* < 0.05 was considered statistically significant for all analyses. All analyses were conducted with SPSS (Version 21.0 for Windows).

## Results

**FIGURE 1:**
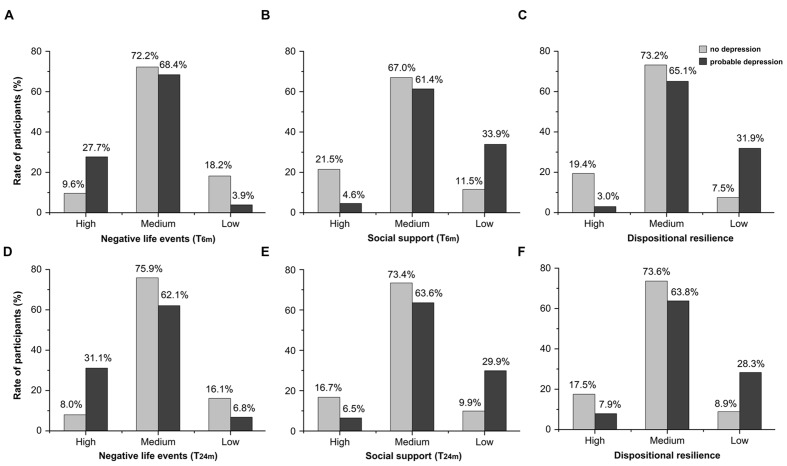
**(A–C):** the rates of participants at T_6m_ according to depressive symptoms (probable depression and no depression) and different groups (high group, medium group and low group) on negative life events (T_6m_), social support (T_6m_), and dispositional resilience. **(D–F)**: the rates of participants at T_30m_ according to depressive symptoms (probable depression and no depression) and different groups (high group, medium group, and low group) on negative life events (T_24m_), social support (T_24m_), and dispositional resilience.

**FIGURE 2:**
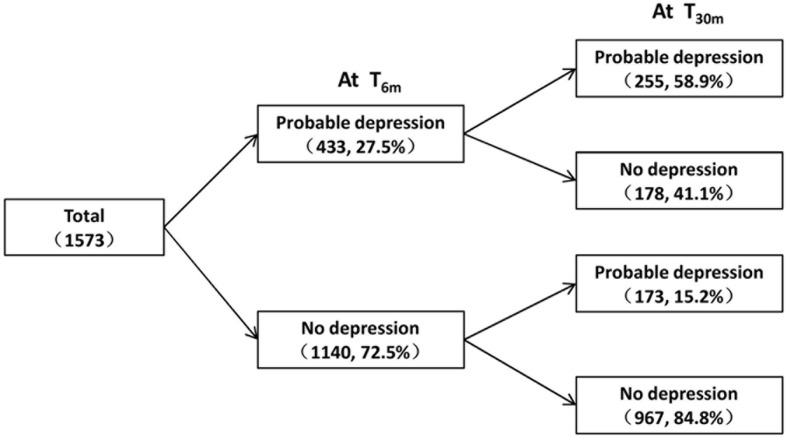
**Change patterns of depressive symptoms after Wenchuan earthquake**.

Demographic characteristics, earthquake exposure, and psychosocial measurements by depression status are shown in **Table 1** and **Figure 1**. The prevalence rates of probable depression at T_6m_ and T_30m_ were 27.5 and 27.2%, indicating that depressive symptoms of the whole sample were stable over time post-earthquake. As shown in **Figure 2**, 178 participants who met the criterion for probable clinical depression at T_6m_ had no significant depressive symptoms at T_30m_ (recovery group), and 173 participants who did not show significant depressive symptoms at T_6m_ developed probable clinical depression at T_30m_ (delayed-onset group). Compared to those who had no depression at both time points, the recovery group experienced more negative life events (*t* = 8.24, *p* < 0.001, Cohen’s *d* = 0.64) at T_6m_, and the delayed-onset group had less social support (*t* = -6.36, *p* < 0.001, Cohen’s *d* = 0.52) and more negative life events(*t* = 8.65, *p* < 0.001, Cohen’s *d* = 0.59) at T_24m_. Besides, independent-samples *t*-tests also showed that females had more negative life events (*t* = 3.26, *p* < 0.001, Cohen’s *d* = 0.16) and lower dispositional resilience (*t* = -3.84, *p* < 0.001, Cohen’s *d* = 0.20) than males at T_6m_. Adolescents who were only child had more social support (*t* = 3.64, *p* < 0.001, Cohen’s *d* = 0.25) at T_24m_ than those with siblings.

**Table 1:** Demographics and earthquake exposure at two time points by depression status (*n* = 1,573).

Variable	6 months after earthquake		30 months after earthquake	
	No dep	Probable dep	No dep	Probable dep
N	1140	433	1145	428
Gender				
Male	552 (48.4%)	168 (38.8%)	561 (49.0%)	159 (37.1%)
Female	588 (51.6%)	265 (61.2%)	584 (51.0%)	269 (62.9%)
No. of children in the family				
1	939 (82.4%)	364 (84.1%)	971 (84.8%)	332 (77.6%)
≥2	201 (17.6%)	69 (15.9%)	174 (15.2%)	96 (22.4%)
Family member injured or killed/missing				
No	853 (74.8%)	294 (67.9%)	860 (75.1%)	287 (67.1%)
Injured	153 (13.4%)	71 (16.4%)	152 (13.3%)	72 (16.8%)
Killed/missing	134 (11.8%)	68 (15.7%)	133 (11.6%)	69 (16.1%)
House damage				
No	119 (10.4%)	40 (9.2%)	118 (10.3%)	41 (9.6%)
Medium	545 (47.8%)	203 (46.9%)	541 (47.2%)	207 (48.4%)
Severe	476 (41.8%)	190 (43.9%)	486 (42.4%)	180 (42.0%)
Property loss				
No	126 (11.0%)	35 (8.1%)	123 (10.7%)	38 (8.9%)
Medium	775 (68.0%)	299 (69.0%)	783 (68.4%)	291 (68.0%)
Severe	239 (21.0%)	99 (22.9%)	239 (20.9%)	99 (23.1%)
Directly witnessed the disaster				
No	448 (39.3%)	155 (35.8%)	451 (39.4%)	152 (35.5%)
Yes	692 (60.7%)	278 (64.2%)	694 (60.6%)	276 (64.5%)

*No dep, No depression; Probable dep, Probable depression.*

**Table 2** shows results from multivariate logistic regression analyses. At T_6m_, adolescents who were females (OR 1.39, 95% CI 1.07–1.80, *p* = 0.01), directly witnessed tragic scenes during the earthquake (OR 1.33, 95% CI 1.01–1.73, *p* = 0.04) and experienced more post-earthquake negative life events (OR for medium vs. low = 3.21, 95% CI 1.84–5.59, *p* < 0.001; OR for high vs. low = 9.72, 95% CI 5.26–17.99, *p* < 0.001) had higher likelihood of developing depression. In addition, individuals who reported less social support (OR for medium vs. low = 0.34, 95% CI 0.25–0.46, *p* < 0.001; OR for high vs. low = 0.10, 95% CI 0.06–0.17, *p* < 0.001) and lower dispositional resilience (OR for medium vs. low = 0.27, 95% CI 0.20–0.38, *p* < 0.001; OR for high vs. low = 0.06, 95% CI 0.03–0.12, *p* < 0.001) had a higher risk of depression.

**Table 2:** Multivariate logistic regression models predicting probable depression at T_6m_ and T_30m_ (*n* = 1,573).

Variable	Probable dep at T_6m_		Probable dep at T_30m_	
	OR (95% CI)	*p*	Adjusted OR (95% CI)^a^	*p*
Gender
Male	1		1	
Female	**1.39 (1.07–1.80)**	**0.01**	**1.41 (1.06–1.87)**	**0.02**
No. of children in the family				
1	1		1	
≥2	0.82 (0.58–1.15)	0.26	**1.86 (1.31–2.64)**	**0.001**
Family member injured or killed/missing				
No	1		1	
Injured	1.16 (0.81–1.66)	0.42	1.22 (0.82–1.82)	0.31
Killed/missing	1.21 (0.82–1.76)	0.33	1.30 (0.86–1.97)	0.22
House damage				
No	1		1	
Medium	0.83 (0.52–1.33)	0.45	0.95 (0.57–1.58)	0.84
Severe	0.81 (0.50–1.32)	0.41	0.80 (0.47–1.37)	0.42
Property loss				
No	1		1	
Medium	1.35 (0.84–2.16)	0.22	1.28 (0.76–2.15)	0.35
Severe	1.08 (0.62–1.88)	0.80	1.20 (0.65–2.20)	0.56
Directly witnessed the disaster				
No	1		1	
Yes	**1.33 (1.01–1.73)**	**0.04**	1.21 (0.91–1.63)	0.20
Negative life events (T_6m_)				
Low	1		1	
Medium	**3.21 (1.84–5.59)**	**<0.001**	1.11 (0.69–1.79)	0.66
High	**9.72 (5.26–17.99)**	**<0.001**	1.24(0.69–2.21)	0.47
Negative life events (T_24m_)				
Low	–	–	1	
Medium	–	–	1.38 (0.84–2.26)	0.20
High	–	–	**5.73 (3.23–10.16)**	**<0.001**
Social support (T_6m_)				
Low	1		1	
Medium	**0.34 (0.25–0.46)**	**<0.001**	1.02 (0.70–1.47)	0.94
High	**0.10 (0.06–0.17)**	**<0.001**	1.80 (0.96–3.15)	0.12
Social support (T_24m_)				
Low	–	–	1	
Medium	–	–	**0.36 (0.25–0.53)**	**<0.001**
High	–	–	**0.19 (0.10–0.35)**	**<0.001**
Dispositional resilience (T_6m_)				
Low	1		1	
Medium	**0.27 (0.20–0.38)**	**<0.001**	0.79 (0.45–1.40)	0.42
High	**0.06 (0.03–0.12)**	**<0.001**	**0.67 (0.46–0.96)**	**0.03**

*^a^Adjusting for depressive symptoms at T_6m_;*

*Probable dep, Probable depression; Data are given as odds ratio (95% confidence interval).*

*Bolded values indicate the significant results.*

At T_30m_, after adjusting for depressive symptoms at T_6m_, the probability of developing depression would increase if individuals were females (OR 1.41, 95% CI 1.06–1.87, *p* = 0.02) and have siblings (OR 1.86, 95% CI 1.31–2.64, *p* = 0.001). The likelihood of developing depression was greater for those having more negative life events (OR for high vs. low = 5.73, 95% CI 3.23–10.16, *p* < 0.001) and less social support (OR for medium vs. low = 0.36, 95% CI 0.25–0.53, *p* < 0.001; OR for high vs. low = 0.19, 95% CI 0.10–0.35, *p* < 0.001) at T_24m_. Meanwhile, adolescents having lower dispositional resilience (OR for high vs. low = 0.67, 95% CI 0.46–0.96, *p* = 0.03) were more likely to develop depression. Moreover, earthquake exposure, negative life events (T_6m_), and social support (T_6m_) could not predict depressive symptom at T_30m_.

## Discussion

To the best of our knowledge, there have been very few studies to longitudinally investigate the changes of depressive symptoms in a large sample of adolescent survivors over a period of more than 2 years after the Wenchuan earthquake. The prevalence of probable depression at T_6m_ and T_30m_ were, respectively, 27.5 and 27.2%, meaning that depressive symptoms were common even after 30 months of the earthquake. The estimates in our study were relatively higher in relation to other studies that investigated depressive symptoms among children and adolescents exposed to the Wenchuan earthquake. For example, in Liu et al. (2011), 330 children aged 8–11 years were assessed by Trauma Symptom Checklist for Children-Alternate Version (TSCC-A) and reported the rates of depressive symptoms to be 14.5 and 16.1% at 6 and 12 months post-earthquake, respectively. Using Children’s Depression Inventory (CDI), Jia et al. (2013) investigated 596 children aged between 8 and 16 years and revealed that 13.9 and 13.5% of them, respectively, endorsing depression at 15- and 30-months post-earthquake. The variation in prevalence rates might be due to differences in depression measurement and sample characteristics across studies. In addition, we also found that the recovery group experienced more negative life events at T_6m_, and the delayed-onset group had less social support and more negative life events at T_24m_ in relation to those who had no depression, which may partly demonstrate why some people only had significant depressive symptoms in the early stage after the earthquake and the others only had in the late.

Multivariate logistic regression showed that there were different patterns for demographic variables including gender and sibling number in predicting the risk of depression at T_6m_ and T_30m_. Female gender was related with increased risk of depressive symptoms at both time points. Such gender effect on posttraumatic mental health problems/disorders has been reported in a large number of studies (Udwin et al., 2000; Furr et al., 2010; Fan et al., 2011; Adams et al., 2014). Our data also showed that females had notably higher level of negative life events at T_6m_ as well as lower level of dispositional resilience than males, which may partially explain the observed strong and long-lasing predictive effect of female gender on depressive symptoms. Other potential explanations are that females in relation to males are more responsive to stresses, which may put them at higher risk for mental problems (Weiss et al., 1999). Furthermore, being only child was found to be associated with lower risk of depression at T_30m_, but not with the risk of depression at T_6m_. Comparisons of negative life events, social support and dispositional resilience between only children and children-with-siblings showed that significant between-group difference only emerged for social support at T_24m_. Inferably, at the early stage after earthquake, no matter a child being only child or not, he/she could receive much care and support from family members or other closed ones, but as time went by, children with siblings might not be given as much care and support as those given to only children. This may explain why being only child or not was only a significant predictor for depressive symptoms at T_30m_, but not for depressive symptoms at T_6m_.

The current study found greater severity of earthquake exposure and more negative life events were associated with higher risk of depressive symptoms after the earthquake. However, the predictive effects of earthquake exposure and negative life events at T_6m_ only held for depressive symptoms at T_6m_, but not for depressive symptoms at T_30m_. Besides, we found that negative life events at T_24m_ could predict depressive symptoms at T_30m_. These results together suggested that earthquake exposure and negative life events may have strong but short-term effects on adolescent survivors’ depressive symptoms. Prior studies also reported that the effect of earthquake exposure on survivors’ mental status may abate with time (Ye et al., 2014). As time went by, individuals might gradually adapt to changes caused by disasters through utilizing external or internal psychosocial resources. As for negative life events, previous study indicated that they have an enduring predictive effect on subsequent depression (Franko et al., 2004), which is inconsistent with our results. This discrepancy may be caused by different measurements of negative life events across studies.

Our results also showed that more social support at T_6m_ could concurrently predict lower risk of depressive symptoms at T_6m_, but could not predict depressive symptoms at T_30m_, while social support at T_24m_ could predict the latter. As the literature (Paykel, 1994; Galambos et al., 2004; Pietrzak et al., 2009; Xu et al., 2013), these findings suggested that social support may be as a protective factor against depression after experiencing a disaster or trauma. Social support might protect a person against depression by buffering the negative impact of traumatic experiences and subsequent daily-life adversity on his/her psychological well-being. However, our findings also indicated that the buffering effect of initial social support at one particular time point may gradually fade away with the passage of time. The similar pattern has also been observed in previous studies (Cook and Bickman, 1990; Altindag et al., 2005). For instance, Altindag et al. (2005) investigated the changes of PTSD symptoms from 1 to 13 months among earthquake survivors in Turkey and found that social support were related with the onset of PTSD within a short period of time but could not predict the chronicity of PTSD over a long time course. These results highlight the importance of providing long-term social support after the disasters. Meanwhile, it is also necessary to help the survivors improve their capability to perceive social support.

The current study also showed that dispositional resilience could negatively predict depressive symptoms at both time points. This is consistent with previous studies (Green et al., 2010; Min et al., 2012). For example, Green et al. (2010) found that veterans with higher resilience were less likely to develop depressive symptomology, even after controlling for age, gender, minority status, trauma exposure, and PTSD diagnosis. Proposed explanations were that resilient individuals were confident of their personal strengths in the face of misfortune (Pyszczynski et al., 2004), employed effective coping to reduce distress (Rajkumar et al., 2008), used positive affect to bounce back from stressors (Tugade and Fredrickson, 2004), used positive thinking to reappraise their loss (Shiota and Levenson, 2012), and established hope and optimism for the future (Boldor et al., 2012).

Several limitations of this study should be noted. First, due to the suddenness and unpredictability of the earthquake, we did not collect data on participants’ depressive symptoms pre-earthquake. Such data would help us better understand the impact of the earthquake on adolescents’ mental health status and make more accurate estimates of those developing depressive symptoms after the earthquake. Second, owing to the large sample size, we did not conduct clinical interviews to diagnose depression and data were conducted through self-report questionnaires, which might be limited by reporting bias. Nevertheless, all measures in our study have been widely used in epidemiological studies in China. Third, our sample was selected from one of the worst-affected areas, as such the results may not be generalizable to the whole population of adolescents affected by the Wenchuan earthquake.

## Conclusion

Our study made a unique contribution to the literature by longitudinally investigating depressive symptoms and related predictors in a large sample of adolescent survivors over a long period of 2 years and a half after the Wenchuan earthquake. The major findings were fourfold: (1) the prevalence of probable depression among adolescent survivors maintained relatively stable over time; (2) female gender was related with higher risk of depressive symptoms at both T_6m_ and T_30m_, while being only-child could only predict higher risk of depressive symptoms at T_30m_; (3) earthquake exposure, negative life events, and social support may have strong but short-term effects on adolescents’ depressive symptoms; (4) dispositional resilience was evidenced as a relatively stable negative predictor for depressive symptoms. These findings have important implications in screening and identifying adolescents at high risk for depression after a disaster, thereby appropriate and targeted mental health interventions can be provided.

## Author Contributions

XS analyzed the data and wrote up the first draft of the manuscript. NY, YZ, and FG helped analyze the data and assist in writing up the draft. FF designed the study and helped to interpret the data. All authors approved the final version of the manuscript.

## Conflict of Interest Statement

The authors declare that the research was conducted in the absence of any commercial or financial relationships that could be construed as a potential conflict of interest.
